# Neural network‐derived Potts models for structure‐based protein design using backbone atomic coordinates and tertiary motifs

**DOI:** 10.1002/pro.4554

**Published:** 2023-02-01

**Authors:** Alex J. Li, Mindren Lu, Israel Desta, Vikram Sundar, Gevorg Grigoryan, Amy E. Keating

**Affiliations:** ^1^ Department of Chemistry Massachusetts Institute of Technology Cambridge Massachusetts USA; ^2^ Department of Electrical Engineering and Computer Science Massachusetts Institute of Technology Cambridge Massachusetts USA; ^3^ Department of Biological Engineering Massachusetts Institute of Technology Cambridge Massachusetts USA; ^4^ Department of Biology Massachusetts Institute of Technology Cambridge Massachusetts USA; ^5^ Computational and Systems Biology Program Massachusetts Institute of Technology Cambridge Massachusetts USA; ^6^ Department of Computer Science Dartmouth College Hanover New Hampshire USA; ^7^ Koch Institute for Integrative Cancer Research Massachusetts Institute of Technology Cambridge Massachusetts USA

**Keywords:** AlphaFold, Bcl‐2, energy based modeling, graph neural networks, Potts models, structure‐based protein design, tertiary motifs

## Abstract

Designing novel proteins to perform desired functions, such as binding or catalysis, is a major goal in synthetic biology. A variety of computational approaches can aid in this task. An energy‐based framework rooted in the sequence‐structure statistics of tertiary motifs (TERMs) can be used for sequence design on predefined backbones. Neural network models that use backbone coordinate‐derived features provide another way to design new proteins. In this work, we combine the two methods to make neural structure‐based models more suitable for protein design. Specifically, we supplement backbone‐coordinate features with TERM‐derived data, as inputs, and we generate energy functions as outputs. We present two architectures that generate Potts models over the sequence space: TERMinator, which uses both TERM‐based and coordinate‐based information, and COORDinator, which uses only coordinate‐based information. Using these two models, we demonstrate that TERMs can be utilized to improve native sequence recovery performance of neural models. Furthermore, we demonstrate that sequences designed by TERMinator are predicted to fold to their target structures by AlphaFold. Finally, we show that both TERMinator and COORDinator learn notions of energetics, and these methods can be fine‐tuned on experimental data to improve predictions. Our results suggest that using TERM‐based and coordinate‐based features together may be beneficial for protein design and that structure‐based neural models that produce Potts energy tables have utility for flexible applications in protein science.

## 1 INTRODUCTION

A fundamental goal of computational protein design is to identify a protein sequence that can adopt a specified structure and exhibit a desired function. Computational methods have guided the design of enzymes (Siegel et al., 2010) and small proteins that bind to the spike protein of SARS‐CoV‐2 and inhibit infection (Cao et al., 2020). Traditionally, protein design has been dominated by physics‐based models such as Rosetta (Leman et al., 2020). However, the computational complexities of modeling molecular interactions at scale require judicious approximations of molecular physics. Accumulated error from these approximations results in inaccuracies, such that physics‐based models typically must be coupled with multiple rounds of expensive experimental trial‐and‐error to meet design objectives (Cao et al., 2020; Frappier & Keating, 2021; Ingraham et al., 2019).

Recently, deep‐learning methods have been applied to computational protein design, as reviewed by several authors (Defresne et al., 2021; Gao et al., 2020; Ovchinnikov & Huang, 2021). Exciting new approaches to de novo design are emerging that simultaneously generate sequence and structure. An example is the work of Anishchenko et al. (2021), who used a deep‐learning “hallucination” method to produce folded proteins without specifying a target structure. The model relies on the transform‐restrained Rosetta model trRosetta, which was developed for structure prediction. Designing a sequence to adopt a known or previously specified structure is also an important problem with many application areas, and new methods are emerging for this task as well. Such methods typically represent proteins using static coordinates obtained from x‐ray crystallography experiments, although designed backbones can also be used (Ingraham et al., 2019; Jing et al., 2021; Strokach et al., 2020). Norn et al. (2021) demonstrated backpropagation of gradients through trRosetta to generate designed sequences for target backbones that were predicted to have high specificity for the intended structure. Anand et al. (2022) applied a 3D convolutional neural network to select a sequence and accompanying side‐chain conformations (rotamers) for a target backbone, generating all‐atoms designs, and showed excellent experimental agreement of two designed TIM‐barrels with their intended structures.

A promising class of methods for performing design on a known backbone structure, which we consider in this work, uses graph neural networks (GNNs). GNNs model proteins as a network of residues (nodes) and residue‐residue interactions (edges) and demonstrate notable performance on the task of recovering native‐like sequences given known protein backbones, significantly outperforming Rosetta on challenging test sets curated to be structurally distinct from the training data (Dauparas et al., 2022; Ingraham et al., 2019; Jing et al., 2021; Strokach et al., 2020). GNNs typically respect the rotational and translational invariance of protein properties with respect to a coordinate basis and are generated by using the local environment to iteratively refine the features describing a residue or residue‐residue interaction. A GNN model for protein design introduced by Ingraham et al. (2019), the Structured Transformer, was elaborated by Jing et al. (2021), who added geometric vector perceptrons to the GNN framework. Hsu et al. (2022) subsequently retrained the Jing model using AlphaFold‐predicted structures in addition to experimentally solved structures, demonstrating that this improves native sequence recovery on a fixed backbone, as discussed later in this paper. The Ingraham model was also modified by Dauparas et al. (2022), who recently reported experimental structures demonstrating successful design of both monomeric and oligomeric proteins using the method ProteinMPNN.

There are potential pitfalls when using deep‐learning models. Neural networks can suffer from overfitting, a phenomenon in which the networks use a very large number of model parameters to memorize the training data rather than learning more general trends. As neural networks achieve increasingly better performance on native sequence recovery tasks, there is concern that models are becoming “backbone readers,” learning to read crystallographic patterns in published protein structures rather than extracting more general properties of protein structure that can be applied in new design problems. Dauparas et al. (2022) demonstrated a technique for mitigating this risk by incorporating coordinate noise during training.

Another drawback of current models arises from the form of output of the model. Natural language processing has spurred many advances in deep learning that have been subsequently applied in protein design. These frameworks generate sequences for input structures using a conditional per‐residue learned probability distribution, which can be sampled from to generate new sequences either linearly (e.g., from N‐terminus to C‐terminus) (Ingraham et al., 2019; Jing et al., 2021) or in a random order (Dauparas et al., 2022). However, this type of sequential sequence generation is difficult to adapt to certain common protein design tasks. For example, such a distribution does not naturally allow for investigation of specific residue‐residue interactions. It is also costly and not intuitive to perform property‐based optimization, such as designing proteins with net positive charge or high predicted solubility. More broadly, such models do not provide a picture of the sequence‐function landscape of a protein structure, that is, a map of how fitness changes as the sequence is varied, which is a property of great interest to protein scientists.

In light of these limitations, statistical models that capture sequence‐structure relationships offer potential solutions. An approach focused on Tertiary Motifs (TERMs) has shown success quantifying relationships between sequence and structure (Zhou et al., 2020). TERMs are compact structural units that recur frequently in many unrelated proteins, and Mackenzie et al. showed that a large portion of protein structure space can be described using TERMs (Mackenzie et al., 2016). Information in TERMs can be used to define a statistical energy potential for a protein by searching for the closest matches to substructures in the protein across the PDB and quantifying the sequence statistics of the resulting matches. Such a potential function, in the form of a Potts model, can be used in design and has been implemented as a procedure known as dTERMen (Zhou et al., 2020). This procedure introduces a degree of backbone flexibility into design because there is permissiveness in the structural matching of TERMs. The overall approach and the dTERMen method in particular have proven valuable. Statistical models based on TERMs can detect incorrect regions in predicted structures (Zheng et al., 2015), predict mutational changes in protein stability (Zheng & Grigoryan, 2017), and be applied directly to protein design (Frappier et al., 2019; Zhou et al., 2020).

In light of the successes of dTERMen, we reasoned that features from this approach could be applied to build better GNN models for protein design. Specifically, introducing a structure featurization that does not rely on a fixed set of backbone coordinates can potentially mitigate the problem of overfitting or coordinate memorization. Additionally, models that output an energy function over sequence variables can be used more flexibly and for a greater number of applications than models that directly output sequences. Potts models can efficiently describe an energy landscape over sequence space using a decomposition into single‐residue and residue‐pair contributions that is convenient and intuitive to structural biologists. Potts models can be used for tasks such as predicting mutational energies, optimizing just a subset of a structure, or sampling protein sequences under constraints (Frappier et al., 2019; Zhou et al., 2020).

In this work, we designed two deep neural networks that generate an energy landscape over sequences on a particular structure: TERMinator, which takes both TERM data and backbone coordinate data as inputs, and COORDinator, which takes only backbone coordinate data. Comparisons of TERMinator and COORDinator show that TERM data are essential for the best performance. We additionally demonstrate that TERMinator designs are predicted by AlphaFold (Jumper et al., 2021) to fold to their target structures. Finally, we present evidence that both TERMinator and COORDinator learn notions of energetics, and we demonstrate that these models can be trained on experimental binding data to improve predictions of binding energies and protein stabilities. Our results suggest that TERMs provide a useful featurization of proteins for deep learning models and that outputting Potts models adapts GNNs for energy‐based tasks.

## 2 RESULTS

### 2.1 Model architecture and training

**FIGURE 1:**
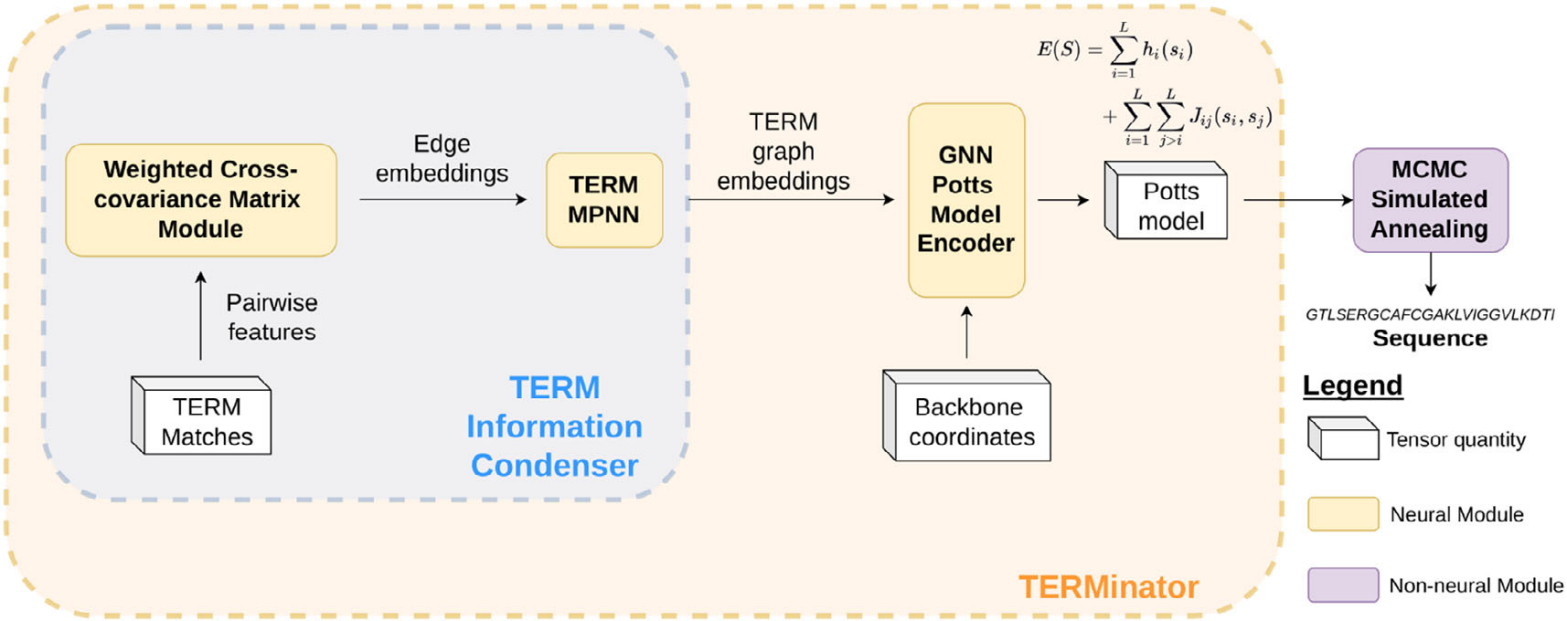
Model architecture. The TERM information condenser extracts information from structural matches to TERMs in the target protein to construct node and edge embeddings. The GNN Potts model encoder takes in TERM data and coordinate features and outputs a Potts model over positional amino acid labels (see Supporting Information for the functional form). We use MCMC simulated annealing to generate optimal sequences given the Potts model.

Our architecture is based on the Structured Transformer from Ingraham et al. (2019), which we adapted to consider new inputs and produce Potts models as outputs. At a high level, TERMinator generates a Potts model by iteratively refining a graph‐based representation of a protein. The network, shown in Figure 1, can be broken into two sections. The first section, the TERM Information Condenser, learns local structure via graph updates on small, local TERM graphs. The second section, the GNN Potts Model Encoder, learns global structure via graph updates over nearest neighbors across the entire chain. Using this global structure graph representation, the model outputs a Potts model over positional residue labels. COORDinator operates in a similar fashion, but consists only of the GNN Potts Model encoder. More information about the network architecture can be found in Section 4.2 and in Supporting Information, including Figure S1.

TERMinator uses experimentally determined protein structures as training data to learn sequence‐structure relationships. The model extends the Structured Transformer and takes a structure as input to generates a Potts model over sequence variables as output; the loss function is the negative log of the composite pseudo‐likelihood of the native sequence on a given structure, as described in Section 4. We trained TERMinator on two datasets: a previously curated single‐chain dataset split using CATH topologies (Ingraham et al., 2019) (the “Ingraham Dataset”), and a new multi‐chain dataset split using sequence redundancy (the “Multichain Dataset”). Our use of a multi‐chain training set was motivated by our desire to apply these methods to the design of protein complexes and protein interfaces, which are lacking in the single‐chain dataset. Protein interfaces can have different structures and residue compositions from single folded chains. Furthermore, the Ingraham Dataset includes protein chains that are taken out of their context in a complex, which presents the model with structures that would not fold as presented in the absence of a binding partner. Details regarding model design, as well as the generation and composition of the two datasets, are discussed further in Section 4.

### 2.2 TERMs improve model performance on native sequence recovery tasks

For both datasets, we trained TERMinator and COORDinator, alongside several ablated versions, to understand how the different modules and input features affect performance on native sequence recovery (NSR). Native sequence recovery measures the percent residue identity between the original protein sequence and the sequence obtained by optimizing the structure‐specific TERMinator‐generated Potts model. Results are shown in Table 1 and detailed descriptions of each model ablation are in Supporting Information. Our best models improve upon the Structured GNN and also on the related GVP‐GNN protein‐design model of Jing et al., as demonstrated by performance on the Ingraham Dataset (Ingraham et al., 2019; Jing et al., 2021).

**TABLE 1:** Ablation studies of TERMinator

Model version	Ingraham NSR (%)	Multichain NSR (%)
*TERMinator*: TIC + GPME	42.40 ± 0.02	46.16 ± 0.15
Linearize TIC	41.39 ± 0.16	44.56 ± 0.04
Ablate TERM MPNN	41.89 ± 0.11	45.42 ± 0.15
Ablate coordinate‐based features, retain *k*‐NN graph	34.19 ± 0.05	37.89 ± 0.35
Ablate GPME	32.38 ± 1.65	36.62 ± 0.06
*COORDinator*: GPME alone (no TERM information)	40.25 ± 0.17	43.46 ± 0.15
dTERMen*	24.32	26.61
GVP‐GNN (Jing et al., 2021)	40.2	‐
Structured GNN (Ingraham et al., 2019) (as reported by Jing et al. (2021))	37.3	‐

*Note*: Native sequence recovery is listed as mean ± SD on median performance of triplicate train/test runs on the same data split, where triplicate data are available. dTERMen* is a handicapped version of dTERMen run without near‐backbone TERMs. Italicized models indicate models released in this paper. TIC, TERM information condenser; GPME, GNN Potts model encoder: see Section 4.

Ablation studies indicate that although TERM‐based features and coordinate‐based features are largely redundant, neither serves as a full replacement for the other. COORDinator, which is trained purely on coordinate data and outputs a Potts energy function, achieves NSR on the Ingraham Dataset of 40.3%, outperforming the Structured GNN. Similarly, training on TERM data with no coordinate information (but with the benefit of the global *k*‐NN graph, an inherently fuzzy feature) also achieves a respectable NSR of 34.2%. However, the full TERMinator model outperforms both of these models, as well as similar published models (Ingraham et al., 2019; Jing et al., 2021), on NSR for the Ingraham Dataset. Similar trends in performance were observed for the Multichain Dataset.

When we remove coordinate data as inputs, TERMinator effectively acts as a better version of dTERMen (Zhou et al., 2020). Ablated TERMinator and dTERMen* (a handicapped version of dTERMen with no access to near‐backbone TERMs) share access to essentially the same TERM input data (see Supporting Information for a more detailed discussion), but the learned model makes better use of this data, with an NSR of 34.2% vs. 24.3% on the Ingraham Dataset and 37.9% vs. 26.6% on the Multichain Dataset for TERMinator vs. dTERMen*.

### 2.3 TERMinator designs physically realistic sequences

#### 2.3.1 TERMinator position‐wise mutations are physically realistic

**FIGURE 2:**
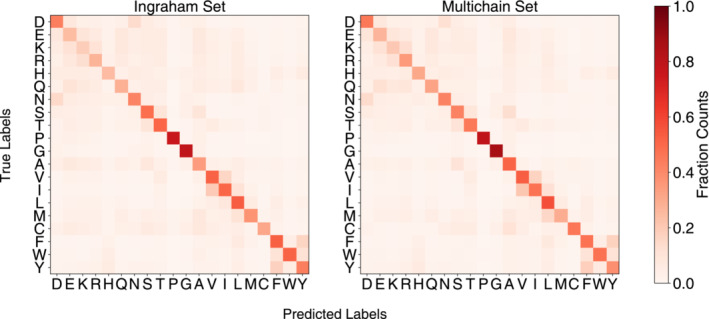
Confusion matrices comparing position‐wise predicted residue identity (*x* axis) versus native residue identity (*y* axis). Values are normalized by the number of times the native residue occurs in the test set. Values report aggregate performance by the full TERMinator model across triplicate runs, on both the Ingraham and multichain datasets.

To better understand the performance of TERMinator, we examined what pointwise substitutions it makes when it does not predict the native residue in NSR tasks. This was done by examining the amino‐acid confusion matrix, where the *x*‐axis represents the predicted residue identity, and the *y*‐axis represents the native residue identity. Representative matrices are shown in Figure 2. The strong diagonal in the matrix reflects the high overall native sequence recovery. Glycine (G), which is small and uniquely flexible, and proline (P), which lacks an amide proton and is conformationally constrained, are particularly well recovered. Interestingly, the substitutions the model makes are physically realistic. We see confusion within the EKR block, which contains charged amino acids glutamate (E), lysine (K), and arginine (R). The switch of charge polarity is potentially attributable to the model reversing the direction of salt bridges. Other blocks include the following: ST, with highly similar serine (S) and threonine (T) hydroxyl sidechains; VI, with sterically similar branched aliphatic valine (V) and isoleucine (I) sidechains; FWY, encompassing large hydrophobic aromatic residues phenylalanine (F), tryptophan (W), and tyrosine (Y); and DN, with isosteric aspartate and asparagine. These substitutions are highly plausible and suggest that TERMinator learns physicochemically realistic representations of proteins.

#### 2.3.2 TERMinator occasionally generates pathological, low‐complexity designs

Although the confusion matrix reflects position‐wise prediction trends, it does not capture sequence‐wide properties. Upon examining TERMinator‐designed sequences, we noticed that a nontrivial portion of designed sequences were dominated by one or a few residues. To detect such cases, we defined a complexity metric that counts the number of possible arrangements of residue labels in a designed sequence (Wootton & Federhen, 1993) (see Section 4 for details). As shown in Figure S2, the complexity distributions for native and TERMinator‐designed sequences differed significantly: no native sequence in the Ingraham test set had a complexity value less than 1.67, whereas 198 of 1120 TERMinator designs had complexity score less than this value. Based on this, we classified designs with complexity <1.67 as “low complexity.”

For low‐complexity cases, we reran MCMC simulated annealing on the respective energy landscapes but added a complexity‐based penalty during sequence design. After applying this penalty, the complexity of the designed sequences improved significantly, with no sequence having a complexity lower than 1.67 (Figure S2). Among the redesigned sequences, we observed a 3.7% mean increase in NSR (Figure S3). Given these results, we adapted our protocol to include redesign of any low‐complexity sequences using the low‐complexity penalty.

#### 2.3.3 TERMinator‐designed sequences are predicted to fold to the corresponding native structure

**FIGURE 3:**
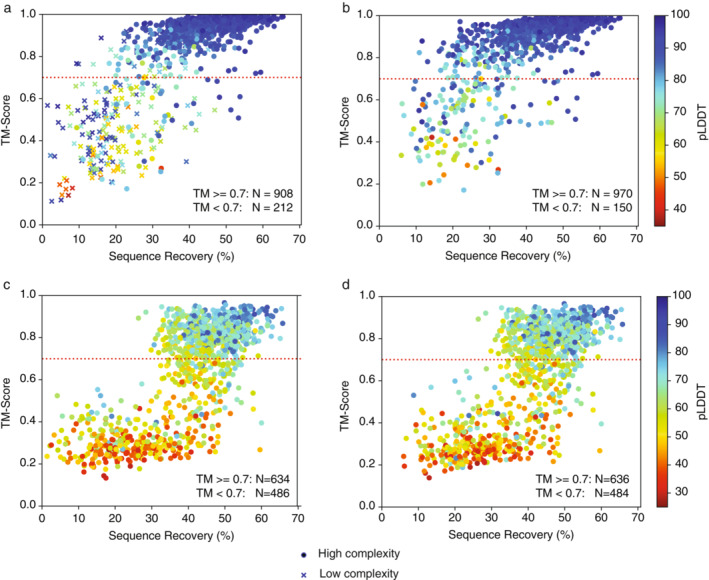
Fold‐specificity analysis on Ingraham test‐set cases. (a) TM‐scores for AlphaFold predictions for TERMinator‐designed sequences versus native sequence recovery. Low‐complexity sequences are indicated using crosses. (b) Same plot as (a) after redesigning low‐complexity sequences using a low‐complexity penalty. (c) Same plot as (a) but for random sequences with NSR values matching those in (a). (d) Same as (c) but for random sequences with NSR values matching those in (b). In all panels, the AlphaFold predicted local distance difference test (pLDDT), which corresponds to confidence in prediction quality, is represented by the hue of the data point, as indicated in the heatmap key.

We evaluated whether TERMinator‐designed sequences are predicted to fold to the target structure, a metric of designed‐protein fold specificity. We used AlphaFold (Jumper et al., 2021) to predict the structure of TERMinator designs for the Ingraham test‐set structures, all of which are single‐chain structures. To evaluate performance, we used template modeling scores, or TM‐scores (Zhang & Skolnick, 2004; Zhang & Skolnick, 2005), which quantify the structural similarity of the predicted structure to the native structure. A TM‐score of 0.7 corresponds to a ≥90% probability that the predicted structure is in the same fold family as the native structure. As expected, at high NSR, predicted structures matched the native fold very well. However, TERMinator also designed sequences with low NSR that were predicted to fold to the intended structure (Figure 3a,b).

When 198 low‐complexity designs were redesigned using the low‐complexity penalty, we observed a 0.17 mean increase in TM‐score, with 62 structures crossing the TM‐score threshold of 0.7 (Figure 3a versus Figure 3b; Figure S4). Notably, including the high‐complexity cases, only 150 cases out of 1120 had TM‐scores below 0.7, and only 70 had TM‐scores below 0.5. Overall, the structures that AlphaFold predicted with high confidence generally matched the input structure fold; using the predicted local distance difference test (pLDDT) value as a metric for AlphaFold confidence, 924 of 975 predicted structures with pLDDT greater than 0.8 had a TM‐score greater than 0.7. For the 106 cases in Figure 3 with NSR <30% and TM‐score >0.7, 47 had <10 sequences in the MSA and 21 had no sequences in the MSA, from any of the three databases that AlphaFold searches. Figure S5 shows five examples of low‐NSR sequences that were successfully folded by AlphaFold without any MSA.

To determine how much fold specificity TERMinator encodes beyond NSR alone, we created a control based on random sequences. We generated sequences with the same NSR values as the TERMinator designs, but with residues matching at random positions. Randomly generated sequences were folded using AlphaFold and TM‐scores for the resulting structures were calculated with respect to the native chains (Figure 3c,d). For random sequences with NSR greater than 50%, there is enough information that AlphaFold can often predict the corresponding fold, even though pLDDT is lower than for TERMinator‐designed sequences. In contrast, for TERMinator‐designed sequences, the high‐NSR examples have a higher success rate, and many low‐NSR sequences are predicted to adopt the target structure, as shown in Figure 3b. These results, as well as the confusion matrices in Figure 2, suggest that the TERMinator potential can be used to provide physicochemically realistic sequences.

### 2.4 TERMinator and COORDinator can be used for analysis of protein energetics

TERMinator is not explicitly trained to model energetics. However, the output of the model takes the form of an energy function, and we investigated the correlation of TERMinator pseudo‐energies with experimental observables including peptide binding affinities and protein resistance to proteolysis, which is a proxy for thermodynamic stability.

#### 2.4.1 Binding affinities for Bcl‐2 proteins

Following a benchmark performed with dTERMen by Frappier et al. (2019), we investigated whether Potts models generated by TERMinator and COORDinator could predict binding energies for Bcl‐2 family protein‐peptide complexes. Bcl‐2 proteins are of particular interest due to their role in controlling apoptosis, a form of programmed cell death, and their overexpression in many cancers (Singh et al., 2019). We compared our results to earlier work using other structure‐based energy evaluations.

**FIGURE 4:**
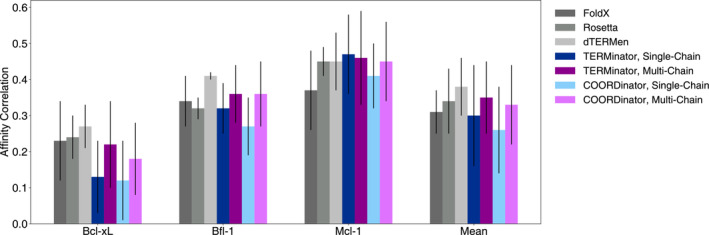
Structure‐based prediction of binding energies for Bcl‐2 protein complexes (Frappier et al., 2019; Jenson et al., 2018). For TERMinator and COORDinator, the Pearson correlation between the predicted and experimental binding energies was calculated and is reported as the mean and SD over all structural templates and triplicate training runs, the variance arises from variable performance across structures, not replicates. The performance data for FoldX and Rosetta were taken from previous work (Frappier et al., 2019), and the performance of dTERMen was recomputed with the latest version of the model as of January 2022. Individual values can be found in Table S1.

We used 13,084 affinity measurements across three anti‐apoptotic Bcl‐2 family proteins that bind to *α*‐helical peptides of roughly 20 residues in length: Bcl‐x_L_, Mcl‐1, and Bfl‐1 (Jenson et al., 2018). Although the three proteins share the same fold, they have only 24% pairwise sequence similarity with one another (Foight & Keating, 2015). We measured the Pearson correlation between TERMinator or COORDinator predicted energies and the experimental affinity measurements. The most relevant comparisons are shown in Figure 4, with the full results in Table S1.

The performance of TERMinator and COORDinator is on par with existing methods FoldX, Rosetta, and dTERMen that predict energies from structural data (Frappier et al., 2019). Using TERMs in addition to backbone coordinates had only a small effect, which was not statistically significant for 2 of the 3 families (Bfl‐1, Mcl‐1). The models trained on the Multichain Dataset performed better than those trained on the Ingraham Dataset in all but one example (Mcl‐1 predictions using TERMinator), which is unsurprising because the task involves predicting the stability of a two‐protein complex (Table S1). Using paired *t*‐tests to compare models, and including data for all three proteins, we obtained *p* = 0.0011 between the multi‐chain and single‐chain forms of TERMinator and *p* = 4.68 × 10^−7^ between the two forms of COORDinator.

#### 2.4.2 Stability of de novo mini‐proteins

**FIGURE 5:**
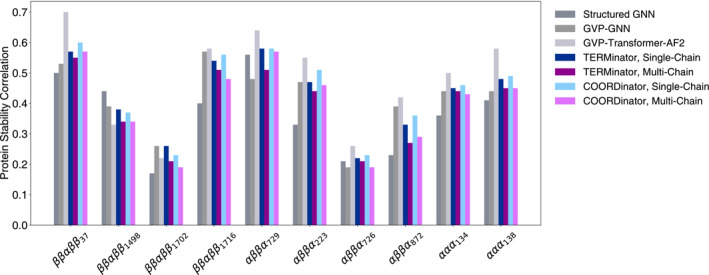
Structure‐based prediction of mutational effects on designed protein domain stability (Rocklin et al., 2017). The performance of TERMinator and COORDinator models is given as the average across triplicate training runs of each model, with SDs all ≤0.04 omitted for clarity. The performance of other models discussed in the text is reproduced from the literature: Structured GNN from Ingraham et al. (2019); GVP‐GNN from Jing et al. (2021); GVP‐transformer‐AF2 from Hsu et al. (2022). Individual values are available in Table S2.

Next, we evaluated TERMinator and COORDinator on a dataset from Rocklin et al. (2017). In this work, the authors performed deep mutational scans for a set of de novo designed mini‐proteins and three natural protein domains, Yap65, villin, and Pin1, using a high‐throughput proteolysis assay that was shown to provide good estimates of protein stability (Rocklin et al., 2017). We compared the results of single‐chain and multi‐chain TERMinator and COORDinator with the flexible‐backbone version of the Structured Transformer of Ingraham et al. (2019), the GVP‐GNN model of Jing et al. (2021), and the GVP‐Transformer‐AF2 model of Hsu et al. (2022). These models were not all trained on the same data. The Structured Transformer and our models were trained on the Ingraham Dataset, which was derived from the CATH 4.2 annotations. The GVP models were trained using a similar dataset derived from CATH 4.3 annotations, and the GVP‐Transformer‐AF2 model used an additional 12 million structures predicted using AlphaFold (Jumper et al., 2021) as training data. We computed the Pearson correlation between TERMinator or COORDinator pseudo‐energies and proteolysis‐based stability values. We give the results for the 10 proteins benchmarked in previous work in Figure 5, and in Table S2 we report performance on seven additional proteins in Rocklin et al. (2017) dataset that were not included in previously published tests (Hsu et al., 2022).

TERMinator and COORDinator both showed performance similar to other state‐of‐the‐art models, aside from the GVP‐Transformer‐AF2 model, which performs the best but is also trained on 1000× more structures. Given the improvement in performance observed when the base GVP‐GNN was augmented with additional training data using AlphaFold predictions, we expect that our models, which already outperform the GVP‐GNN, may see a similar boost in performance after training data augmentation. Our single‐chain models performed better than our multi‐chain models, consistent with the test involving single‐chain proteins. However, the addition of TERMs had little impact on the models' predictive power for this mini‐protein stability test.

#### 2.4.3 TERMinator can be fine‐tuned to improve energy‐based protein analysis

Motivated by the results of our initial benchmarking, we tested whether incorporating experimental binding energy data in training could improve the performance of TERMinator and COORDinator on energy‐based protein analysis tasks. We refined both models via a process known as fine‐tuning, where a model that is already trained on a larger, more general dataset is updated in a retraining process that uses a smaller but more tailored dataset.

We used the Bcl‐2 binding affinity data (Jenson et al., 2018) to fine‐tune TERMinator and COORDinator. During fine‐tuning, we trained the model to minimize a balance between the negative log composite pseudo‐likelihood and the negative Pearson correlation between the experimentally measured binding affinities and predicted Potts model energies (see Section 4).

Figure 4 shows that TERMinator and COORDinator perform best for Mcl‐1 binding data, followed by Bfl‐1 and Bcl‐x_L_. For our fine‐tuning test, we chose to use Mcl‐1 binding data as the training set, as it had the best performance to begin with. We used Bcl‐x_L_ data as the validation set; this allowed us to stop training before overfitting. Bfl‐1 data served as our test set, on which we report results. We performed fine‐tuning using both the single‐chain and multi‐chain models, and both TERMinator and COORDinator. The data in Table 2 show that fine‐tuning is successful at improving affinity correlation performance on the held‐out Bfl‐1 family test set by an average of 0.19 points across all models.

**TABLE 2:** TERMinator and COORDinator performance before and after fine‐tuning

Model version	Baseline			Fine‐tuned		
	Bfl‐1 *r*	Rocklin *r*	Test NSR %	Bfl‐1 *r*	Rocklin *r*	Test NSR %
TERMinator, single‐chain	0.32 ± 0.07	0.45 ± 0.12	42.40 ± 0.02	0.51 ± 0.03	0.53 ± 0.11	35.67 ± 1.60
TERMinator, multi‐chain	0.36 ± 0.08	0.42 ± 0.12	46.16 ± 0.15	0.52 ± 0.05	0.51 ± 0.11	40.95 ± 1.75
COORDinator, single‐chain	0.27 ± 0.08	0.46 ± 0.13	40.25 ± 0.17	0.51 ± 0.03	0.54 ± 0.11	33.52 ± 0.99
COORDinator, multi‐chain	0.36 ± 0.09	0.42 ± 0.14	43.46 ± 0.15	0.51 ± 0.04	0.49 ± 0.11	38.28 ± 0.42

*Note*: Bfl‐1 performance is reported as an average and SD over all Bfl‐1 complex templates across triplicate training runs. Performance on the Rocklin dataset (Rocklin et al., 2017) is reported across triplicate runs over all 17 structures in the dataset. Native sequence recovery (NSR) is reported as the mean percentage test set recovery across median performance on triplicate runs on the Ingraham and multichain datasets for the single‐chain and multi‐chain models, respectively. Per‐fold performance on the Rocklin dataset can be found in Table S3.

We also evaluated the models that were fine‐tuned using Mcl‐1 and Bcl‐x_L_ protein binding data on the Rocklin dataset. This represents a stringent test of generalizability, because the Rocklin dataset contains de novo designed structures that have no relation to the Bcl‐2 family. In Table 2, we show that fine‐tuning improved performance on this test by an average of 0.08 points across all models. This improvement suggests that we successfully fine‐tuned TERMinator and COORDinator in a way that generates Potts models that are more physically realistic and potentially broadly useful. For the single‐chain models, numerical improvement results for each individual fold are in Table S3.

We evaluated the fine‐tuned single‐chain and multi‐chain models on the original Ingraham and Multichain test sets, respectively, and report NSR performance in Table 2. The NSR values for the multi‐chain models each dropped about 5%, while the NSR on single‐chain models fell about 7%–9%. Thus, although performance on energy benchmarks improved after fine‐tuning, it came at a cost to NSR. Note that the low‐complexity cases obtained from fine‐tuned versions of all single‐chain and multi‐chain models were redesigned with the same protocol discussed in Section 4.4.

### 2.5 Investigation of TERMinator Potts models reveals disconnect between sequence‐based and energetics‐based metrics

As discussed above, we noticed that TERMinator and COORDinator Potts models sometimes assigned low energies to low‐complexity sequences (e.g., long repeating stretches of the same residue label). Although this did not occur for the majority of structures, and we were able to avoid this behavior by imposing a penalty on low‐complexity sequences during design, we were curious about why the learned Potts models favored these clearly incorrect solutions.

Because dTERMen does not exhibit the same preference for low‐complexity sequences, we compared the single‐residue and residue‐pair terms in the dTERMen vs. TERMinator and COORDinator Potts models. The scatterplots in Figure S6 reveal two clusters corresponding to the self‐energy parameters (orange) and the pair‐energy parameters (blue). Notably, TERMinator puts more weight into the pair‐energy parameters, whereas dTERMen puts more weight into the self‐energy parameters.

We speculated that the relatively larger pair‐energy weights in TERMinator may allow certain Potts models to have pathological low‐energy, low‐complexity minima that arise from spurious second‐order effects. Hence, we sought to encourage TERMinator to place more weight on self energies in training. Due to the much greater number of pair‐energy parameters compared to self‐energy parameters, we decided to train TERMinator with a penalty on the vector norm of all learned parameters (see Section 4), as a Potts model with larger self energies but smaller pair energies would have a smaller norm than a Potts model with the opposite characteristics. As shown in Figure S6, this penalty reduced the magnitude of both pair and self‐energy weights. We performed design using the new model, without any low‐complexity penalty. Notably, using the retrained model, not only did the complexity of the sequences designed for test‐set structures increase (Figure S7), but the median NSR also increased by as much as 2.5% (Table 3).

**TABLE 3:** TERMinator and COORDinator performance with and without the Potts model parameter norm penalty

Model version	Ingraham NSR (%)	Multichain NSR (%)	Bcl‐2 *r*	Rocklin *r*
TERMinator, no norm penalty	42.40 ± 0.02	46.16 ± 0.15	0.35 ± 0.10	0.45 ± 0.12
TERMinator, with norm penalty	44.52 ± 0.21	48.64 ± 0.02	0.33 ± 0.15	0.41 ± 0.11
COORDinator, no norm penalty	40.25 ± 0.17	43.46 ± 0.15	0.33 ± 0.11	0.46 ± 0.13
COORDinator, with norm penalty	42.24 ± 0.18	46.02 ± 0.12	0.32 ± 0.14	0.40 ± 0.11

*Note*: Native sequence recovery is listed as mean ± SD over median performance for triplicate train/test runs on the same data split. For the Bcl‐2 results, we report the performance averaged across all families (Mcl‐1, Bfl‐1, Bcl‐x_L_) using the multichain version of each model. For the Rocklin results, we report the performance averaged across all 17 structures in the dataset using the single‐chain version of each model.

We expected that the rebalanced Potts model would also improve the performance of TERMinator on the Bcl‐2 and Rocklin binding and stability benchmarks. However, this was not the case. As shown in Table 3, models trained using the penalty did not give improvements on either task.

We performed a similar analysis on the output Potts models before and after fine‐tuning, as shown in Figure S8. While the qualitative features of the energy distribution did not change significantly, the magnitude of the energy values decreased, similar to what was observed upon regularization. We also note the performance trend for finetuning is the reverse of that of the norm penalty: while performance on the energetics benchmarks improved, NSR also decreased.

## 3 DISCUSSION

### 3.1 Ingraham vs. multichain dataset

Our study builds on the pioneering formulation of protein design using the Structured Transformer, as proposed by Ingraham et al. (2019). We have explored several modifications and extensions of this work, including training the model to learn a Potts model of protein energy as a function of sequence variables and incorporating a “fuzzier” representation of sequence‐structure relationships using TERMs. Another change was our use of different training data for modeling protein complexes. Other recent work by Dauparas et al. (2022) has also expanded the Structured Transformer using different approaches, for example, investigating different backbone featurizations and decoding schemes.

To train TERMinator and COORDinator, we used two datasets. The Ingraham Dataset was generated by splitting all proteins in the nonredundant CATH 4.2 dataset into their individual chains, and then partitioning the chains into three sets such that no CATH fold was present in more than one set. This process included many protein chains without the binding partners (other protein chains) that are present in experimentally studied complexes. Because a binding partner may affect the protein conformation or structural environment of residues in a single‐chain training example, such cases of missing contextual information may reinforce nonphysical sequence‐structure relationships, thereby compromising the ability of a model to describe binding interfaces correctly.

We generated our Multichain Dataset by splitting a nonredundant set of the PDB into three sets such that no individual chain in the test set shared more than 50% sequence similarity with any chain in the training or validation datasets. Although this led to a less rigorous structural split among the three sets than in the Ingraham Dataset, because training and test examples may have the same CATH fold, this strategy allowed the model to learn about all protein chains in the context of their binding partners.

TERMinator and COORDinator performed better on the Bcl‐2 benchmark when trained on the multichain dataset. This is expected because the Bcl‐2 dataset involves a binding interface between a peptide and a target protein. In contrast, all structures in the Rocklin benchmark are single‐chain structures, and TERMinator and COORDinator performed better on this test when trained on single‐chain data, possibly benefiting from the stricter training split and improved generalization.

### 3.2 TERMinator vs. COORDinator

TERMinator and COORDinator, while built from the same modules, differ in that TERMinator has access to both TERM and coordinate data whereas COORDinator uses coordinate data alone. Notably, TERMinator achieves higher NSR than COORDinator (Tables 1 and 3), which suggests that on tasks where recapitulating the native sequence is a good metric, TERMs provide nonredundant information. This may be because prespecifying TERM matches provides explicit information about the tolerated structural flexibility and its associated sequence statistics in a given TERM context. In principle, this flexibility can be learned, and indeed the good performance of COORDinator suggests that the network accomplishes this to a significant degree.

However, mining TERMs is relatively time‐expensive, at roughly 4 min per residue. While our studies indicate that TERMinator outperforms COORDinator on NSR, TERMs do not consistently improve energetics on either the Bcl‐2 or Rocklin benchmarks. Therefore, in applications that require the evaluation of many structures, mining TERMs may not be worth the additional overhead cost. In such cases, COORDinator may be of greater use, as it performs well on the tested benchmarks without requiring TERMs. COORDinator has the potential to enable more facile high‐throughput in silico energetics analyses: energy tables can be generated in seconds, with an average inference time of 73 μs per residue using 1 NVIDIA V100 GPU and 465 μs per residue on CPU.

### 3.3 Benefits of Potts models for representing energy landscapes

Given that TERMinator is an energy‐based model, it can be applied to any question that can be formulated in an energy‐based framework, for example, questions related to binding affinity or mutational effects. Additionally, the energy function is decomposable into component interactions, enabling investigation of and optimization over specific residues or reside‐residue interactions. The Potts model form also allows for various other tasks such as constrained optimization, as done with dTERMen and other Potts models (Frappier et al., 2019; Grigoryan et al., 2009; Jenson et al., 2018). This opens up many possibilities for design, especially if we consider the additional potential for model improvement via fine‐tuning and additional training data.

### 3.4 Reflecting on metrics in protein design

During our investigations of model performance, we noticed several phenomena that raise interesting questions regarding Potts model design and the suitability of using NSR as a proxy metric for protein design.

First, although the median NSR of TERMinator and COORDinator is high, compared to other methods, the model occasionally produces low‐information sequences with very low native sequence identity. Notably, the structures that give rise to low‐complexity sequences are, themselves, often of low structural complexity. For example, the Ingraham test set contains single chains from helical bundles and coiled‐coil oligomers that are relatively featureless alpha helices. In our NSR tests, sequences for these helices were designed without the appropriate oligomeric context. While such structures' native sequences are not low‐information, the decontextualized input helps explain why TERMinator‐generated sequences have this quality.

To investigate this phenomenon in more detail, we examined the distribution of energetic parameters of TERMinator relative to statistically derived dTERMen. After observing that the TERMinator Potts model distributes energetic parameters differently from dTERMen Potts models, we attempted to prevent pathological cases by implementing a penalty on the vector norm across all energetic parameters. Although this increased NSR across the board, no performance increase was seen on energetics‐based tasks. Given that protein stability and binding energy are much more important than NSR for most protein design tasks, this casts doubt on the suitability of NSR as a metric when performing method development.

Another disconnect between NSR and desired model properties was observed when we folded TERMinator‐designed sequences using AlphaFold (Jumper et al., 2021). A number of designed sequences had relatively low NSR values but high TM‐scores, reinforcing the point that sequences with low similarity to the native sequence can still fold to the desired structure. Given that variation on the native sequence may be required for many applications (for example, increasing or decreasing the immunogenicity of a protein, or increasing its solubility while maintaining its structure), selecting for protein design models based on high NSR may limit design flexibility. A similar criticism applies to selecting models based on low model perplexity.

Finally, we noted that when we fine‐tuned the model using protein‐peptide binding data, which improved performance on energy prediction tasks, the NSR decreased. This provides an indication that NSR may be a nonoptimal measure of the utility of a learned energy function for protein design tasks. The native sequence is not likely to be the sequence that will give the most stable fold of a given structure, even though this sequence may be the sequence that fits best onto a precisely specified set of crystallographic coordinates. Most protein domains have many closely related homologous sequences that can adopt closely related structures, and mutational studies reveal that proteins can routinely be stabilized by point mutations or by more extensive redesign, for example, by consensus‐sequence redesign (Sternke et al., 2019). Such stabilizing changes may be accompanied by small adjustments of local structure. Machine‐learning methods trained to associate specific backbone coordinates with a given sequence may over‐train on these precise residue arrangements. We believe that introducing other information can reduce these biases that arise from over‐specified coordinates. Indeed, Dauparas et al. (2022) recently made a related observation about the ProteinMPNN model, specifically that native sequence recovery decreases even as AlphaFold‐predicted foldability increases, as noise is introduced into training coordinates.

### 3.5 Promise of improving models with additional data

TERMinator and COORDinator are promising models because of the capacity to improve predictions given additional data. One avenue for improving the performance of protein design models is to allow them to learn directly from experimental data. There are a limited number of experimental datasets large enough for machine learning purposes, but our proof‐of‐concept shows that fine‐tuning TERMinator on the Bcl‐2 affinity benchmark improved performance not only for Bcl‐2 binding predictions but also for the unrelated Rocklin proteolysis stability benchmark. This indicates the capacity for model generalization and the potential of making larger improvements by incorporating other data.

Additionally, with the advent of AlphaFold (Jumper et al., 2021), the use of predicted structures as training data provides a promising way to boost the performance of protein design models. Scaling the size of the TERMinator model and expanding the training data using predicted structures (as done in Hsu et al. (2022)) could lead to a significant performance enhancements and greater generalizability.

## 4 MATERIALS AND METHODS

### 4.1 Datasets

We trained TERMinator on two datasets: a published single‐chain dataset (Ingraham et al., 2019), and a new multi‐chain dataset. For every protein, we described the structure using a set of Tertiary Motifs (TERMs), which are small, local‐in‐space fragments of proteins that recur frequently across unrelated proteins (Mackenzie et al., 2016). The selected TERMs consisted of sequence‐local singleton TERMs (up to 6 contiguous residues) and sequence nonlocal pair TERMs (usually 2 interacting 3‐residue segments). Notably, TERMs are selected such that every residue has at least one TERM describing it. For each TERM, we performed substructure lookup (Zhou et al., 2020) against a nonredundant database generated from the Protein Data Bank (PDB) on January 22, 2019. Self‐matches with the protein itself were discarded. For each match, we recorded the sequence along with several geometric features of the backbone. For more details about the recorded TERM data, see Supporting Information.

Each dataset was split into training, validation, and test subsets, as described below. The model was allowed to learn from the training data. The validation data were used to determine when to terminate training so as to avoid over‐fitting. Model performance was evaluated using the test dataset.

#### 4.1.1 Multichain dataset

We constructed the multichain dataset in the following manner. First, the PDB on January 22, 2019 was filtered for x‐ray structures primarily containing protein components, such that no chain in any given structure contained more than 500 residues. Then, membrane proteins were removed, and structures were filtered for resolution better than 2.6 Å. This resulted in 115,583 proteins. This set of proteins was made nonredundant using usearch (Edgar, 2010) by clustering structures based on sequence similarity and then sampling one representative structure from each cluster. This resulted in 14,747 protein structures. This set was then randomly split 90/10 into a training subset and a validation subset, both of which were filtered to remove any structures that contained fewer than 30 residues. This resulted in 13,083 training‐set structures and 1455 validation‐set structures. Using BLAST+ (Camacho et al., 2009), we calculate the chain‐wise sequence redundancy by running all‐against‐all BLAST with an *E*‐value criterion of 10^−6^, taking the highest sequence redundancy hit per chain, and computing the mean of the resulting values. This yields a multichain training set sequence redundancy of 57.5%. For reference, the same metric for the Ingraham training set is 44.9%.

The test data were constructed from all structures in the PDB deposited between January 22, 2019 and May 12, 2021. We computed sequence redundancy across all chains in the newly deposited structures by running BLAST+ (Camacho et al., 2009) with an *E*‐value criterion of 10^−6^ against the training set, validation set, and all structures used in the TERM match lookup database. We then discarded structures for which at least one chain within the structure had >50% computed sequence redundancy. The filtered dataset was then made nonredundant by using usearch (Edgar, 2010) and sampling one representative structure from each cluster; all structures that contained fewer than 30 residues were removed. This resulted in 1105 test‐set structures.

### 4.2 Architecture

#### 4.2.1 TERM information condenser

TERMinator decomposes a target structure into TERMs and mines structural matches to these TERMs (as described above in Section 4.1). We represent the TERMs as bidirectional fully connected graphs that include self‐edges. Per TERM, we take the top 50 matches in the PDB with lowest RMSD. Each residue in each TERM match is converted to a set of geometric features describing backbone geometry and residue accessibility along with a one‐hot encoding of the match residue identity. Node embeddings are initially set as an empty zero vector of proper dimensionality. Edge embeddings, representing residue interactions within a TERM, are computed using cross‐covariance features between residues in a TERM. Self‐edges are also represented in this manner.

We feed these preliminary TERM graph embeddings through a neural module called the TERM MPNN, which creates a featurized graph representing each TERM in a protein. Via a series of learnable graph updates, a collection of residue embeddings and edge embeddings per TERM is generated. We then merge all TERM graphs to form a full‐structure graph, taking advantage of the fact that all residues and residue interactions in the structure are covered by at least one TERM. For nodes and edges that are covered by multiple TERMs, the representation for that node or edge is generated by averaging the representations across all covering TERMs.

#### 4.2.2 GNN Potts model encoder

The GNN Potts Model Encoder combines the structure embedding produced by the TERM Information Condenser with target protein backbone coordinate features to produce a Potts model over sequence space. TERMinator uses the coordinate‐based structure embedding presented in Ingraham et al. (2019), and this is fused, via concatenation of features, with the TERM‐based structure embedding from the TERM Information Condenser. Concatenation of the coordinate‐based and TERM‐based embeddings occurs if the node or edge exists in the global *k*‐nearest neighbors graph of residues; otherwise, a zero‐vector of equivalent dimensionality is concatenated to the coordinate‐based embedding. COORDinator uses only the Ingraham coordinate‐based structure embedding.

The initial full‐chain representation is processed by the GNN Potts Model Encoder (see Supporting Information for further details). The GNN Potts Model Encoder is a neural module that is identical to the TERM MPNN in architecture, but which operates on the global *k* nearest‐neighbors (*k*‐NN) graph, including self‐edges. We produce a Potts model from the output of this network by projecting each edge embedding into a matrix of residue‐pair interaction energies, averaging any duplicate bidirectional interaction energies (e.g., interaction *i* → *j* and interaction *j* → *i*). Self‐energies are defined as the diagonal of this matrix (derived from the self‐edge), whereas pair‐energies are defined by the entire matrix.

### 4.3 Training

#### 4.3.1 Loss functions

The primary loss function used was the negative log *composite pseudo‐likelihood* of the native sequence on a given structure, averaged across residue pairs. Composite pseudo‐likelihood is the probability that any pair of interacting residues has the same identity as that pair of residues in the target sequence, given the remainder of the target sequence. Composite pseudo‐likelihood can be defined using the energies described by the Potts model as follows. As stated in Supporting Information section “Potts Model,” a Potts model is defined by two functions: the self‐energy function *E*_*s*_(*R*_*i*_ = *m*) evaluates the energy of residue *i* with identity *m*, and the pair‐energy function *E*_*p*_(*R*_*i*_ = *m*,*R*_*j*_ = *n*) evaluates the energy of residue *i* with identity *m* interacting with residue *j* with identity *n*. From the Potts model, we computed the contextual pairwise energy *E*_*cp*_ for a sequence with residue *i* having identity *m* and residue *j* having identity *n*, provided all other residues *R*_*u*_ with identity *r*_*u*_, as:

$$\begin{array}{l} E_{\mathrm{cp}}\left(R_{i}=m,R_{j}=n,\left\{R_{u}=r_{u}\right\}\right)=\\ \;E_{s}\left(R_{i}=m\right)+E_{s}\left(R_{j}=n\right)\\ \;+E_{p}\left(R_{i}=m,R_{j}=n\right)\\ \;+\sum_{u\neq i,j}\left(E_{p}\left(R_{i}=m,R_{u}=r_{u}\right)+E_{p}(R_{u}=r_{u},R_{j}=n)\right) \end{array}$$

From this energy, we compute the composite pseudo‐likelihood *p*(*R*_*i*_ = *m*,*R*_*j*_ = *n*,{*R*_*u*_ = *r*_*u*_}):

$$\begin{array}{l} p\left(R_{i}=m,R_{j}=n,\left\{R_{u}=r_{u}\right\}\right)=\\ \;\frac{\exp\left[-E_{\mathrm{cp}}\left(R_{i}=m,R_{j}=n,\left\{R_{u}=r_{u}\right\}\right)\right]}{\sum_{k,l\in A}\exp\left[-E_{\mathrm{cp}}\left(R_{i}=k,R_{j}=l,\left\{R_{u}=r_{u}\right\}\right)\right]} \end{array}$$

where A represents the set of all residue identities.

During general model training, the loss function was the negative log composite pseudo‐likelihood averaged across residue pairs.

The Potts model norm penalty takes the form

$${ℒ}_{\mathrm{norm}}=\frac{1}{L}{\left[\sum_{i=1}^{L}\sum_{m}E_{s}{\left(R_{i}=m\right)}^{2}+\sum_{i=1}^{L}\sum_{\begin{array}{l} 0<j\leq L\\ j\neq i \end{array}}\sum_{m,n}E_{p}(R_{i}=m,R_{j}=n){}^{2}\right]}^{1/2}$$

This is equal to the normalized L2 norm of all energies in a given Potts model. To train models with this norm penalty as discussed in Section 2.5, we used a weighted sum of the negative log composite pseudo‐likelihood and ${ℒ}_{\mathrm{norm}}$ as the loss function. Using a logarithmic hyperparameter search, we empirically found that weighting these two terms equally led to the best NSR performance on the validation set; this choice was then used for training all evaluated models.

The fine‐tuning objective used the negative Pearson correlation. Consider *N* sequences in a binding affinity dataset. For the *i*th data point, let the experimental binding value be *e*_*i*_ and the predicted binding value be *p*_*i*_. We sought to minimize the negated Pearson correlation, or equivalently maximize the Pearson correlation, resulting in

$${ℒ}_{\mathrm{cor}}=-\frac{\sum_{i=1}^{N}\left(e_{i}-\bar{e}\right)\left(p_{i}-\bar{p}\right)}{\sqrt{\sum_{i=1}^{N}{\left(e_{i}-\bar{e}\right)}^{2}}\sqrt{\sum_{i=1}^{N}{\left(p_{i}-\bar{p}\right)}^{2}}}$$

To fine‐tune our models, we used a weighted sum of the negative log composite pseudo‐likelihood and ${ℒ}_{\mathrm{cor}}$ as the new loss function. Using a logarithmic hyperparameter search, we found that placing 100 times more weight on ${ℒ}_{\mathrm{cor}}$ gave the best compromise between substantial improvements on the Bcl‐2 validation set (Bcl‐x_L_) and modest loss of NSR performance on the Ingraham and Multichain datasets. This choice was used for all fine‐tuning.

#### 4.3.2 Training scheme

We trained each model for 100 epochs with early validation stopping, using the Noam optimizer as reported in Vaswani et al. (2017) based on a model dimensionality of 128. We used a semi‐shuffle batching method which operates in the following fashion: all proteins are first sorted according to their number of TERM residues, then partitioned into partitions of size 500. Within each partition, the order of the proteins is fully shuffled. Then, variable‐size batches are constructed starting with the protein with the fewest number of TERM residues by incrementally adding proteins to a batch as long as the batch has less than 55,000 TERM residues, after which a new batch is started and generated in similar fashion. For COORDinator, we perform the same process but add to the batch while it has less than 6000 residues. After all batches are constructed, the order of batches is shuffled fully before training begins. This process was repeated after every epoch.

The intuition behind this training scheme is to achieve a balance between full shuffling and compute efficiency. We sought to sort proteins of similar length together in order to prevent wasted computation on padding values. However, training on fixed mini‐batches may lead to poor training convergence, so we also wanted mini‐batches to have variability between training epochs. Thus, we opted to shuffle and batch proteins with other similar‐length proteins.

### 4.4 Sequence design

To design sequences, given the Potts model for a structure, we used Markov Chain Monte Carlo (MCMC) processes for global optimization. For each Potts model, we sampled 100 sequences via MCMC‐simulated annealing, running 10^6^ cycles per run while reducing the temperature from *kT* = 1 to *kT* = 0.1. The lowest energy sequence was then taken as the designed sequence.

If a designed sequence was designated as low‐complexity (as described in 2.3, see Equation 4 of Wootton and Federhen (1993)), we redesigned it using a low‐complexity penalty. Specifically, we computed the number of unique arrangements of the amino‐acid labels in sequence *S* as:

$$\Omega \left(S\right)=\frac{L!}{\prod_{i=1}^{20}n_{i}\left(S\right)!}$$

where *n*_*i*_ is the number of times amino acid *i* occurs in *S*. We then defined the complexity of *S* as:

$$K\left(S\right)=\frac{1}{L}\log\left(\Omega \left(S\right)\right)$$

and if it was below 1.67, we labeled the sequence as low‐complexity and added a penalty term to the Potts energy *E*_*Potts*_ to generate a composite score:

$$E_{\mathrm{new}}\left(S\right)=E_{\text{Potts}}\left(S\right)-a\cdot \log\left(\Omega \left(S\right)\right)$$

which was used in MCMC simulated annealing. We empirically found *a* = 1 to work well. We ran redesign MCMC by generating 10 sequences, running 2 × 10^6^ cycles for each while reducing the temperature from *kT* = 10 to *kT* = 0.1. We chose the lowest‐energy sequence (according to *E*_*new*_) as the final design.

### 4.5 AlphaFold folding

The sequences designed by TERMinator for the Ingraham test‐set structures were folded with AlphaFold (Jumper et al., 2021) using the full‐database parameter and default settings in monomer mode. Folding failed for a single case (Chain A of 3CNX) due to a failure in featurizing potential templates. This case was rerun on ColabFold (Mirdita et al., 2022), which utilized a protein database with 70% sequence identity filter for searching for templates and the MMseq2 (Steinegger & Söding, 2017) sequence search tool. After folding, the TM‐Score (Zhang & Skolnick, 2004; Zhang & Skolnick, 2005) of the model with the highest pLDDT was calculated against the native structure.

Randomized sequences were generated by retaining the native residue identity at randomly selected positions and mutating the remaining positions to a randomly selected non‐native residue. More specifically, (1) the number of residues to set as native was determined based on the NSR of the TERMinator design (rounded up to the nearest integer), (2) the positions to retain as native were selected randomly, in the new semi‐random sequence, and (3) the rest of the residues were assigned at random from the 19 residues not equal to the native residue. This resulted in randomized sequences with native‐sequence identities that matched those of the corresponding TERMinator designed sequences.

### 4.6 Energetics and fine‐tuning

#### 4.6.1 Bcl‐2 benchmark

As was done with dTERMen in Frappier et al. (2019), we used a dataset of 4488, 4648, and 3948 affinity measurements for BH3 peptides binding to Bcl‐x_L_, Mcl‐1, and Bfl‐1, respectively (Jenson et al., 2018). Multiple structures are available for each protein in complex with different helical peptides. Following Frappier et al. (2019), we used 15, 25, and 6 structures for Bcl‐x_L_, Mcl‐1, and Bfl‐1, respectively, as inputs to generate Potts models using TERMinator or COORDinator. To make binding energy predictions, we summed the Potts model self‐ and pair‐energy contributions for all peptide positions. Per template, we computed the Pearson correlation between these values and the experimental affinity measurements. We then aggregated results per family by computing the mean and SD across templates within a family.

#### 4.6.2 Rocklin benchmark

We used a dataset from Rocklin et al. (2017) that was generated by performing deep mutational scans and using a proteolysis assay to quantify fold stability in high throughput. The authors studied a set of de novo designed mini‐proteins and three natural protein domains, Yap65, villin and Pin1, with PDB IDs 1JMQ, 1VII, and 2M8I (Luh et al., 2013; McKnight et al., 1997; Pires et al., 2001). For each fold, we generated Potts models with TERMinator and COORDinator and used these to score each mutated protein sequence. We then computed the Pearson correlation between the predicted energies and the experimental stability values.

#### 4.6.3 Fine‐tuning

For fine‐tuning, we froze the weights in all layers except the last output layer. By retraining only the output layer, we avoided both overfitting to the new data and erasing what the model had learned about proteins in the earlier layers. We used the Bcl‐2 binding affinity dataset (Jenson et al., 2018) and associated complex structures to fine‐tune the model. We used the Mcl‐1 binding data as training data and the Bcl‐x_L_ data as a validation, to stop training at the appropriate time. As the loss function, we used a mixed objective between the composite pseudolikelihood and the Pearson correlation between the experimentally measured binding affinities and predicted Potts model energies, weighted 1:100 as discussed in the Loss Functions portion of Section 4.3.

## AUTHOR CONTRIBUTIONS

**Alex J. Li:** Conceptualization (supporting); data curation (equal); formal analysis (equal); investigation (equal); methodology (lead); project administration (equal); software (lead); validation (equal); visualization (equal); writing – original draft (equal); writing – review and editing (equal). **Mindren Lu:** Data curation (equal); formal analysis (equal); investigation (equal); methodology (supporting); project administration (supporting); software (supporting); validation (equal); visualization (supporting); writing – original draft (supporting); writing – review and editing (supporting). **Israel Desta:** Data curation (equal); formal analysis (equal); investigation (equal); methodology (supporting); project administration (supporting); validation (equal); visualization (equal); writing – original draft (supporting); writing – review and editing (supporting). **Vikram Sundar:** Investigation (supporting); methodology (supporting); project administration (supporting); software (supporting); validation (supporting); writing – review and editing (supporting). **Gevorg Grigoryan:** Conceptualization (equal); funding acquisition (equal); methodology (supporting); project administration (equal); resources (equal); software (supporting); supervision (supporting). **Amy Keating:** Conceptualization (equal); funding acquisition (equal); methodology (supporting); project administration (equal); resources (equal); supervision (lead); visualization (supporting); writing – original draft (supporting); writing – review and editing (equal).

## Supporting information


**Appendix S1.** Supporting Informationincluding details of the methods, Figures S1 ‐ S8, and Tables S1 ‐ S3.

## Data Availability

All data and open‐source code are provided at https://github.com/alexjli/terminator_public and https://github.com/KeatingLab/terminator.
