# Supplementary material for: Neural network‐derived Potts models for structure‐based protein design using backbone atomic coordinates and tertiary motifs
Source: Protein Sci. 2023 Feb 1;32(2):e4554. doi: 10.1002/pro.4554 (PMC9854172; doi:10.1002/pro.4554)
Supplement: Supplementary file 1 — Appendix S1. Supporting Informationincluding details of the methods, Figures S1 ‐ S8, and Tables S1 ‐ S3. [file PRO-32-e4554-s001.docx]

1. A Potts Model

Our model outputs a Potts model over positional amino acid labels, commonly known as an energy table. A Potts model describes a mapping from sequence *S* of length *L* to energy *E*(*S*) with the functional form

$$E(S)=\sum_{i=1}^{L} h_{i}(s_{i})+\sum_{i=1}^{L} \sum_{j>i}^{L} J_{ij}(s_{i},s_{j})$$

where

- Singleton terms *h_i_*(*s_i_*) describe the energy contribution of position *i* in *S*.
- Pairwise interaction terms *J_ij_*(*s_i_*,*s_j_*) describe the energy contribution from the interaction between positions *i* and *j* in *S*.

In our Potts model, the singleton term takes the form *h_i_*(*s_i_*) = *E_s_*(*R_i_* = *m*), where *E_s_* is a lookup table of energies for placing residue *m* at position *i*. The pairwise interaction term takes the form *J_ij_*(*s_i_*,*s_j_*) = *E_p_*(*R_i_* = *m*,*R_j_* = *n*), where *E_p_* is a lookup table of energies of placing residue *m* at position *i* and residue *n* at position *j*. This functional form is attractive because it can be used to rapidly evaluate the energy of any sequence, and it is easy to optimize via MCMC-based methods.

1. B TERM data

Our goal was to feed into our neural network similar data as would normally be mined as part of dTERMen.^16^ This would enable us to differentiate the limitations of TERM data themselves from the limitations associated with the specific statistical approach in dTERMen. To this end, we modified an in-house version of the dTERMen program with the ability to output TERM match information for all of the motifs used in the standard procedure (as described in Zhou et al.^16^). Briefly, dTERMen defines three types of TERMs in the input structural template: singleton, near-backbone, and pair TERMs. Singleton TERMs are defined around each residue *i* via the contiguous fragment between residues (*i* - *n*) and (*i* + *n*), where *n* is a parameter (*n* = 1 was used in this study). Near-backbone TERMs combine the local backbone around residue *i* (i.e., the singleton fragment) with local backbone fragments around each residue *j* whose backbone is geometrically poised to interfere with amino-acid sidechains at *i*. Finally, pair TERMs are defined around each pair of residues *i* and *j* that are geometrically positioned to affect each other’s amino-acid choice.

As described in Zhou et al.,^16^ it is frequently the case that the full near-backbone TERM around a residue (i.e., the generally multi-segment motif that captures all relevant surrounding backbone fragments) does not contain sufficient structural matches in the database to generate reliable statistics, in which case the dTERMen procedure seeks to optimally partition the overall near-backbone contribution into as few sub-motifs as possible. This step adds considerable search time. We reasoned that a learning-based approach may be better at extracting relevant statistical couplings between residue sites, such that a detailed breakdown of sidechain-to-sidechain versus backbone-to-sidechain coupling statistics may not be necessary. Therefore, we omitted near-backbone TERMs in this study for computational efficiency.

In finding close structural matches, dTERMen uses a motif complexity-based empirical RMSD cutoff (defined in Mackenzie et al. ^17^), with additional settings used to control the minimal and maximal number of matches. In this study, we set these limits to lower values than previously reported ^16^ for computational efficiency. Specifically, the minimal/maximal match counts were 200/500 for singleton TERMs, and 400/500 for pair TERMs. Under these settings, dTERMen takes roughly 4 minutes per residue (single-core, 8GB RAM). The native sequence recovery rate of dTERMen was estimated on the basis of energy tables produced with these settings. As input into the neural-network models, only data from the top 50 TERM matches were used.

For each considered TERM, we output which positions of the structural template it covers along with information on each of its matches in the database of known structures, i.e. the match sequence, best-fit backbone RMSD from the query, backbone *ϕ* and *ψ* values at each residue, and the ”environment” of each residue–a scalar ranging from 0 to 1 that describes how solvent-exposed the residue is (the freedom metric defined in ^19^); see Figure B1.

Additionally, for every residue in a TERM we compute a “contact index” that specifies the sequence distance from a central residue in the TERM. TERMs are constructed either around a single central residue (for a singleton TERM) or a pair of residues (for a pair TERM). We assign central residues an index of 0 and define the contact index for the remaining residues as the directional sequence distance to the closest intra-chain center residue. More specifically, non-central residues closer to the N-terminus than their corresponding central residue are assigned a negative integer contact index, while non-central residues closer to the C-terminus than their corresponding central residue are assigned a positive integer contact index.

1. C Weighted Cross-Covariance Matrix Features

For each pair of residues, we compute the weighted cross-covariance matrix between the residue features across all matches to the TERM, weighted by RMSD. Let *r_i_* represent the RMSD of match *i* of *n* to the TERM. Then, the weight of match *i* is computed as

$$w_{i}=\frac{e^{-r_{i}}}{\sum_{j} e^{-r_{j}}}$$

Consider a TERM edge from residue *a* to residue *b*. For match *i*, residue *a* has a vector of features *m_a_*_,_*_i_* and residue *b* has a vector of features *m_b_*_,_*_i_*. This vector includes the one-hot encoding of the residue identity, sinusoidally encoded torsion angles, RMSD, and environment value. The weighted mean of features for residue *a* and residue *b*, *μ_ma_*_,_*_i_* and *μ_mb_*_,_*_i_* are

$$\mu_{h_{a,i}}=\sum_{i=1}^{n} w_{i}m_{a,i}$$

$$\mu_{h_{b,i}}=\sum_{i=1}^{n} w_{i}m_{b,i}$$

The cross-covariance matrix is then computed as

$$K_{ab}=\sum_{i=1}^{n} w_{i}(m_{a,i}-\mu_{m_{a,i}})(m_{b,i}-\mu_{m_{b,i}})^{T}$$

This matrix is then flattened into a vector and its dimensionality is reduced using a two-layer feedforward network with ReLU activations. This output is used as the edge feature between the two residues of concern in the TERM graph.

1. D TERM MPNN

We define the following notation:

- *h_i_*_,_*_t_*: the embedding for residue *i* in TERM *t*
- *h_i_*_→_*_j_*_,_*_t_*: the embedding for directional edge *i* → *j* in TERM *t*
- *f_n_*,*f_e_*: three-layer dense networks with ReLU activations
- *g_n_*,*g_e_*: two-layer dense networks with ReLU activations
- __: the set of residues in TERM *t*
- *s_i_*_,_*_t_*: the sinusoidal embedding of the contact index of residue *i* in TERM *t*. We multiply the contact index by 500 before performing the sinusoidal embedding, which we find empirically allows for better learning (see positional embedding in Vaswani et al.^33^).
- [;] represents the concatenation operation

The TERM MPNN utilizes alternative edge-update and node-update layers. The update for a directional edge *i* → *j* in TERM *t* is computed as

$$h_{i\to j,t,\text{update}}=\frac{1}{2}[f_{e}([h_{i,t};s_{i,t};h_{i\to j,t};h_{j,t};s_{j,t}])+f_{e}([h_{j,t};s_{j,t};h_{j\to i,t};h_{i,t};s_{i,t}])]$$

And this update is applied as follows:

$$\begin{matrix} h_{i\to j,t}\leftarrow\text{LayerNorm}(h_{i\to j,t}+\text{Dropout}(h_{i\to j,t,\text{update}})) \\ h_{i\to j,t}\leftarrow\text{LayerNorm}(h_{i\to j,t}+\text{Dropout}(g_{e}(h_{i\to j,t}))) \end{matrix}$$

The update for a node is computed as

And this update is applied as follows:

$$\begin{matrix} h_{i,t}\leftarrow\text{LayerNorm}(h_{i,t}+\text{Dropout}(h_{i,t,\text{update}})) \\ h_{i,t}\leftarrow\text{LayerNorm}(h_{i,t}+\text{Dropout}(g_{e}(h_{i,t}))) \end{matrix}$$

The TERM MPNN contains three layers, with each layer containing an edge update followed by a node update. After these updates, all bidirectional edges are merged into undirected edges via taking the mean of the two edge embeddings.

1. E GNN Potts Model Encoder

The GNN Potts Model Encoder is another message-passing network that is identical to the TERM MPNN in architecture but takes in different input features. The GNN Potts Model Encoder operates on a *k*-NN graph rather than a fully-connected graph, meaning node updates are computed over a residue’s *k* nearest neighbors. Additionally, because there is no notion of “contact index” when it comes to global structure, the update function does not take such features as inputs.

Before running message passing, the GNN must stitch together the TERM-based structure embedding and the coordinate-based structure embeddings. Node embeddings for the GNN are computed by concatenating the coordinate-based features from Ingraham et al. ^5^ and the TERM-based features and feeding that vector through a linear layer to compress the vector back to the original dimensionality. Edge embeddings are also formed by computing the coordinate-based edge embedding from Ingraham et al. ^5^, concatenating the corresponding TERM edge embeddings, and feeding that vector through a linear layer to compress the vector back to the original dimensionality. In the case that a TERM edge embedding does not exist for that particular *k*-NN graph edge, a zero-vector of equal dimensionality is used instead.

The edge embeddings derived after message-passing is completed are then projected to a 400-dimensional vector by a feedforward network and reshaped to form a matrix containing interaction energies between pairs of interacting residues. The interaction energy matrices give the pair energies of the Potts model. Due to the inclusion of self-edges in the *k*-NN graph, we can also compute self-energies for the Potts model by taking the diagonal of the self-interaction matrix produced by the self-edge for each residue.

1. F Hyperparameters

During training we shuffle according to the semi-shuffle method described in Methods and Materials. We use partition sizes of 500 and perform variable-sized batching, with the cutoff being 55000 TERM residues for TERMinator and 6000 residues for COORDinator.

The GNN Potts Model Encoder has a hidden dimensionality of 128, and consists of three node update layers and three edge update layers which are interleaved. Each of these layers use message-computation layers *f* which are three-layer dense networks with hidden layer sizes (384, 128, 128), as well as feedfoward layers *g* which are two-layer dense networks with hidden layer sizes (512, 128).

The TERM Information Condenser uses a hidden dimension of 32. The cross-covariance matrix uses a 2-layer feedforward network with hidden layer sizes (128, 32). The TERM MPNN consists of three node update layers and three edge update layers which are interleaved. Each of these layers use message-computation layers *f* which are three-layer dense networks with hidden layer sizes (96, 32, 32), as well as feedfoward layers *g* which are two-layer dense networks with hidden layer sizes (128, 32).

The choice of different hidden dimensions for the TERM Information Condenser and the GNN Potts Model Encoder is largely due to GPU memory issues, as raw TERM data are much larger than coordinate data. Given more compute power, one direction to explore is how the model’s performance is affected by varying the hidden dimensionality of both portions of the network.

Across both networks, we use a rate of 0.1 for all Dropout layers.

1. G Description of Ablated Models

The following list provides a brief description of TERMinator ablation models:

- **TERM Information Condenser + GNN Potts Model Encoder:** All neural modules included.
- **Ablate TERM Information Condenser:** The TERM Information Condenser is reduced to a series of linear transformations.
  - **Ablate TERM MPNN:** Initial singleton and pairwise TERM features are directly passed to the GNN Potts Model Encoder.
- **Ablate GNN Potts Model Encoder:** Outputs of the TERM Information Condenser are embedded on a *k*-NN graph and then projected to form a Potts Model.
- **Ablate Coordinate-based Features, Retain *k*-NN graph:** Coordinate-based features are set to 0. The *k*-NN graph is still retained.
- **GNN Potts Model Encoder Alone (no TERM information):** Outputs of the TERM Information Condenser are set to 0.

*Ablate GNN Potts Model Encoder vs. dTERMen*

In the main text, we claim that the version of TERMinator with the GNN Potts Model Encoder ablated has access to essentially the same features as the version of dTERMen that we call dTERMen*. It is important to acknowledge a few differences in the precise inputs. Regarding TERMinator, while this particular ablation form does not have access to coordinates directly, it does have access to a *k*-NN graph, which dTERMen* does not get. However, when we ablate the GNN Potts Model Encoder, it has no opportunities to perform graph operations over the *k*-NN graph; instead, the *k*-NN graph is only used to restrict pair interactions in the Potts model to those present in the graph. Due to the nature of TERMs being constructed out of small sets of spatially-proximal residues (<7 residues), it is almost always the case that all TERM edges will be included in the *k*-NN graph (in this work, *k* = 30), leading to negligible utilization of the *k*-NN graph itself. On the other hand, dTERMen* used more matches to compute the Potts model than does TERMinator, which was restricted to using the top 50 matches. We also note that the published version of dTERMen uses near-backbone TERMs that were not included for the dTERMen* sequence recovery results reported here, although this also means that TERMinator did not have access to these TERMs either. All things considered, it is reasonable to assume that this ablation of TERMinator and dTERMen* effectively have access to the same types of information, with dTERMen* performing worse despite having access to more matches.

1. H Sequence Complexity Plots
2. I Folding examples
3. J Full Results for Energy-Based Benchmarks

Here, we report the individual values for the performance of TERMinator and COORDinator on the energy-based protein analysis tasks discussed in the Results section of this paper.

1. K Potts Model Parameter Distributions for TERMinator and COORDinator

FIGURE B1 TERM matches information structure.

| * TERM k  list of position indices covered by the TERM (suppose N of them) sequence of match 0; RMSD; N phi values; N psi values; N environment values sequence of match 1; RMSD; N phi values; N psi values; N environment values... |
| --- |

FIGURE E1 TERMinator Submodule Architectures.

FIGURE H1 The complexity distribution, based on the number of unique arrangements of labels, of both native and designed sequences for structures in the Ingraham Dataset test set (N=1120). TERMinator re-designed refers to design using the low-complexity penalty, for low-complexity sequences.

FIGURE H2 The changes in (A) native sequence recovery (NSR), and (B) TM-score, after re-designing the Ingraham test set low-complexity cases with the complexity-based penalty. __ represents the mean change and Δ̃ represents the median change, in both graphs, both in percentage points.

FIGURE H3 Complexity of TERMinator designs on the Ingraham test set with and without the norm-based penalty during training.

FIGURE I1 Predicted structures for TERMinator designs, for examples lacking sequence alignments. Examples of TERMinator designing a protein that adopts the correct fold, at low NSR (<30%), as predicted using AlphaFold (AF). These examples had no MSA for structure predictions. The successfully designed/predicted examples are compared to randomized sequences with the same NSR. Crystal structures are in green in all panels; cyan shows TERMinator sequences (re-designed using the complexity penalty) folded by AF, and orange shows the randomized sequences folded by AF.

| Example | PDB | Design | TERMinator | TERMinator | Randomized | Randomized |
| --- | --- | --- | --- | --- | --- | --- |
| label | ID | NSR (%) | design TM-score | design pLDDT | design TM-score | design pLDDT |
| (A) | 1SKV_A | 18.75 | 0.81 | 87.73 | 0.25 | 50.63 |
| (B) | 1PV0_A | 26.09 | 0.77 | 82.72 | 0.38 | 69.58 |
| (C) | 2C5Z_A | 20.43 | 0.81 | 83.26 | 0.31 | 46.81 |
| (D) | 1XEQ_B | 28.24 | 0.84 | 87.41 | 0.45 | 49.32 |
| (E) | 2ZDI_B | 26.42 | 0.93 | 95.74 | 0.45 | 47.65 |

FIGURE I2 Examples of extreme changes in fold specificity before (magenta) and after (cyan) TERMinator redesign with the complexity penalty are shown here after being aligned (using TMalign) to the native chain (shown in green).

| Example label | PDB ID | TM-score before | TM-score after | NSR before (%) | NSR after (%) |
| --- | --- | --- | --- | --- | --- |
| (A) | 1YUA_A | 0.23 | 0.79 | 27.05 | 31.15 |
| (B) | 1SG7_A | 0.30 | 0.87 | 16.0 | 21.33 |
| (C) | 2HKY_A | 0.26 | 0.84 | 19.38 | 27.91 |
| (D) | 1KVZ_A | 0.26 | 0.88 | 20.56 | 28.04 |
| (E) | 4I0X_L | 0.65 | 0.39 | 25.61 | 23.17 |
| (F) | 1BCC_H | 0.63 | 0.34 | 18.18 | 30.30 |

FIGURE K1 TERMinator and COORDinator Potts model parameters versus dTERMen^*^ Potts model parameters for a representative test case, before and after regularization.

FIGURE K2 TERMinator and COORDinator Potts model parameters versus dTERMen^*^ Potts model parameters for a representative test case, before and after finetuning.

TABLE J1 Affinity correlation performance averaged over all Bcl-2 complex templates.^20,26^ These are the summary values used to generate Figure 4.

|  | Bcl-x_L_ | Bfl-1 | Mcl-1 | Mean |
| --- | --- | --- | --- | --- |
| FoldX | 0.23 ± 0.11 | 0.34 ± 0.07 | 0.37 ± 0.11 | 0.31 ± 0.06 |
| Rosetta | 0.24 ± 0.06 | 0.32 ± 0.03 | 0.45 ± 0.04 | 0.34 ± 0.09 |
| dTERMen | 0.27 ± 0.06 | 0.41 ± 0.01 | 0.45 ± 0.08 | 0.38 ± 0.08 |
| TERMinator, Single-Chain | 0.13 ± 0.10 | 0.32 ± 0.07 | 0.47 ± 0.11 | 0.30 ± 0.14 |
| TERMinator, Multi-Chain | 0.22 ± 0.12 | 0.36 ± 0.08 | 0.46 ± 0.13 | 0.35 ± 0.10 |
| COORDinator, Single-Chain | 0.12 ± 0.11 | 0.27 ± 0.08 | 0.41 ± 0.09 | 0.26 ± 0.12 |
| COORDinator, Multi-Chain | 0.18 ± 0.10 | 0.36 ± 0.09 | 0.45 ± 0.11 | 0.33 ± 0.11 |

TABLE J2 Protein stability correlation performance on a mutational stability dataset of *de novo* small proteins,^28^ including previous work.^5,15^ For all proteins and both TERMinator and COORDinator, the standard deviations across triplicate training runs were ≤ 0.04 and are omitted for clarity. The top half of the table contains the values used to generate Figure 5. The bottom half of the table notes our performance on 7 additional structures in the Rocklin et al. ^28^ dataset for which the other models do not report results.

|  | Structured | GVP- | GVP+ | Single-Chain | Multi-Chain | Single-Chain | Multi-Chain |
| --- | --- | --- | --- | --- | --- | --- | --- |
|  | GNN^5^ | GNN^10^ | AF2^15^ | TERMinator | TERMinator | COORDinator | COORDinator |
| *ββαββ* _37_ | 0.50 | 0.53 | 0.70 | 0.57 | 0.55 | 0.60 | 0.57 |
| *ββαββ*_1498_ | 0.44 | 0.39 | 0.33 | 0.38 | 0.34 | 0.37 | 0.34 |
| *ββαββ*_1702_ | 0.17 | 0.26 | 0.22 | 0.26 | 0.21 | 0.23 | 0.19 |
| *ββαββ*_1716_ | 0.40 | 0.57 | 0.58 | 0.54 | 0.51 | 0.56 | 0.48 |
| *αββα*_779_ | 0.56 | 0.48 | 0.64 | 0.58 | 0.51 | 0.58 | 0.56 |
| *αββα*_223_ | 0.33 | 0.47 | 0.55 | 0.47 | 0.44 | 0.51 | 0.46 |
| *αββα*_726_ | 0.21 | 0.19 | 0.26 | 0.22 | 0.21 | 0.23 | 0.19 |
| *αββα*_872_ | 0.23 | 0.39 | 0.42 | 0.33 | 0.27 | 0.36 | 0.29 |
| *ααα*_134_ | 0.36 | 0.44 | 0.50 | 0.45 | 0.44 | 0.46 | 0.43 |
| *ααα*_138_ | 0.41 | 0.44 | 0.58 | 0.48 | 0.45 | 0.49 | 0.45 |
| Mean | 0.36 | 0.42 | 0.48 | 0.43 | 0.39 | 0.44 | 0.40 |
| *ααα*_142_ |  |  |  | 0.65 | 0.61 | 0.65 | 0.62 |
| *ββαβ*_882_ |  |  |  | 0.61 | 0.56 | 0.60 | 0.55 |
| *ββαβ*_5_ |  |  |  | 0.43 | 0.44 | 0.45 | 0.45 |
| *ββαβ*_15_ |  |  |  | 0.38 | 0.36 | 0.37 | 0.36 |
| 1JMQ |  |  |  | 0.45 | 0.39 | 0.41 | 0.36 |
| 1VII |  |  |  | 0.33 | 0.29 | 0.40 | 0.22 |
| 2M8I |  |  |  | 0.56 | 0.52 | 0.56 | 0.54 |
| Mean (all folds) |  |  |  | 0.45 | 0.42 | 0.46 | 0.42 |

TABLE J3 Protein stability correlation performance on a mutational stability dataset of *de novo* small proteins,^28^ for the GVP-Transformer-AF2 model from Hsu et al.^15^ and the single-chain TERMinator and COORDinator models, both before and after fine-tuning. The top half of the table includes the 10 structures studied in previous work, and the bottom half of the table notes our performance on 7 additional structures from Rocklin et al.^28^ which have not been previously benchmarked.

|  | **GVP+AF2 ^15^** | **Single-Chain** | **TERMinator** | **Single-Chain** | **COORDinator** |
| --- | --- | --- | --- | --- | --- |
|  |  | **TERMinator** | **Fine-Tuned** | **COORDinator** | **Fine-Tuned** |
| *ββαββ* _37_ | 0.70 | 0.57 | 0.64 | 0.60 | 0.66 |
| *ββαββ*_1498_ | 0.33 | 0.38 | 0.50 | 0.37 | 0.50 |
| *ββαββ*_1702_ | 0.22 | 0.26 | 0.29 | 0.23 | 0.27 |
| *ββαββ*_1716_ | 0.58 | 0.54 | 0.63 | 0.56 | 0.64 |
| *αββα*_779_ | 0.64 | 0.58 | 0.68 | 0.58 | 0.69 |
| *αββα*_223_ | 0.55 | 0.47 | 0.59 | 0.51 | 0.60 |
| *αββα*_726_ | 0.26 | 0.22 | 0.43 | 0.23 | 0.44 |
| *αββα*_872_ | 0.42 | 0.33 | 0.53 | 0.36 | 0.56 |
| *ααα*_134_ | 0.50 | 0.45 | 0.46 | 0.46 | 0.50 |
| *ααα*_138_ | 0.58 | 0.48 | 0.50 | 0.49 | 0.52 |
| Mean | 0.48 | 0.43 | 0.53 | 0.44 | 0.54 |
| *ααα*_142_ |  | 0.65 | 0.69 | 0.65 | 0.71 |
| *ββαβ*_882_ |  | 0.61 | 0.62 | 0.60 | 0.63 |
| *ββαβ*_5_ |  | 0.43 | 0.51 | 0.45 | 0.53 |
| *ββαβ*_15_ |  | 0.38 | 0.41 | 0.37 | 0.40 |
| 1JMQ |  | 0.45 | 0.49 | 0.41 | 0.46 |
| 1VII |  | 0.33 | 0.47 | 0.40 | 0.50 |
| 2M8I |  | 0.56 | 0.60 | 0.56 | 0.62 |
| Mean (all folds) |  | 0.45 | 0.53 | 0.46 | 0.54 |
